# Toward an ontology of tobacco, nicotine and vaping products

**DOI:** 10.1111/add.16010

**Published:** 2022-08-15

**Authors:** Sharon Cox, Robert West, Caitlin Notley, Kirstie Soar, Janna Hastings

**Affiliations:** ^1^ Department of Behavioural Science and Health University College London London UK; ^2^ SPECTRUM research consortium; ^3^ Addiction Research Group, Norwich Medical School University of East Anglia Norwich UK; ^4^ Centre for Addictive Behaviours Research London South Bank University London UK; ^5^ Department of Clinical, Educational and Health Psychology University College London London UK; ^6^ Institute for Intelligent Interacting Systems Otto‐von‐Guericke University Magdeburg Germany

**Keywords:** E‐cigarette, nicotine, ontology, tobacco, tobacco product, vape, vaping device

## Abstract

**Background and aims:**

Ontologies are ways of representing information that improve clarity and the ability to connect different data sources. This paper proposes an initial version of an ontology of tobacco, nicotine and vaping products with the aim of reducing ambiguity and confusion in the field.

**Methods:**

Terms related to tobacco, nicotine and vaping products were identified in the research literature and their usage characterised. Basic Formal Ontology was used as a unifying upper‐level ontology to describe the domain, and classes with definitions and labels were developed linking them to this ontology. Labels, definitions and properties were reviewed and revised in an iterative manner until a coherent set of classes was agreed by the authors.

**Results:**

Overlapping, but distinct classes were developed: ‘tobacco‐containing product’, ‘nicotine‐containing product’ and ‘vaping device’. Subclasses of tobacco‐containing products are ‘combustible tobacco‐containing product’, ‘heated tobacco product’ and ‘smokeless tobacco‐containing product’. Subclasses of combustible tobacco‐containing product include ‘cigar’, ‘cigarillo’, ‘bidi’ and ‘cigarette’ with further subclasses including ‘manufactured cigarette’. Manufactured cigarettes have properties that include ‘machine‐smoked nicotine yield’ and ‘machine‐smoked tar yield’. Subclasses of smokeless tobacco product include ‘nasal snuff’, ‘chewing tobacco product’, and ‘oral snuff’ with its subclass ‘snus’. Subclasses of nicotine‐containing product include ‘nicotine lozenge’ and ‘nicotine transdermal patch’. Subclasses of vaping device included ‘electronic vaping device’ with a further subclass, ‘e‐cigarette’. E‐cigarettes have evolved with a complex range of properties including atomiser resistance, battery power, properties of consumables including e‐liquid nicotine concentration and flavourings, and the ontology characterises classes of product accordingly.

**Conclusions:**

Use of an ontology of tobacco, nicotine and vaping products should help reduce ambiguity and confusion in tobacco control research and practice.

## INTRODUCTION

Products containing tobacco are widely used worldwide and their use has been estimated to contribute to more than 7 million deaths per year globally [1]. There are many diverse products that differ in popularity according to geographical region, demographic characteristics, legislation and culture. The products also differ in how they are used, how the tobacco is prepared and their harmfulness to health. Cigarettes contribute to the most deaths [1]. Products containing nicotine, but no other tobacco constituents are also used widely, most commonly in an attempt to stop or reduce cigarette consumption [2, 3]. The advent of non‐tobacco nicotine‐containing products, e‐cigarettes and other vaping devices has added to the range of products that need to be considered to understand and address tobacco‐related harm [2, 3]. We have also seen marketing of tobacco products that are heated, but without being fully combusted. Such a wide variety of products with such varied properties makes it essential to have clarity about the type of product to which one is referring [4, 5, 6]. This paper aims to improve clarity by setting out an initial version of an ontology of products related to tobacco, nicotine and vaping with clearly defined classes and relationships between them.

The rapid evolution of tobacco, nicotine and vaping products has caused problems when it comes to defining and labelling these, and some high profile organisations have struggled to achieve logical consistency in their nomenclature [4, 6, 7, 8]. For example, the Food and Drug Association (FDA) in the United States (US), classifies e‐cigarettes as a ‘tobacco product’ [6, 7]. Unfortunately, this creates two inconsistencies. First of all, the only tobacco constituent in e‐cigarettes is nicotine, and nicotine replacement therapy (NRT) products are not classified as tobacco products because they are regulated as medicinal products by the Center for Drug Evaluation and Research (CDER). Second, many e‐cigarettes do not contain nicotine or any other tobacco constituent or even any psychoactive drug. The World Health Organisation (WHO) similarly classifies as tobacco products ones that have no tobacco constituents [9]. Bloomberg Philanthropies, a major funder of tobacco control research, goes further in labelling e‐cigarettes as ‘flavoured tobacco products’, which at a broad level to those without specific product knowledge conflates and equates them with other types of flavoured combustible products [10].

How tobacco‐related products are labelled influences the conduct and interpretation of scientific research. Lack of clarity around products has led to misunderstanding and disputes over the interpretation of data (e.g. Jarvis *et al*.) [11]. For example, estimates of the prevalence of ‘youth tobacco use’ in the United States have been increased by including e‐cigarettes as a tobacco product, whereas headline figures for adult smoking prevalence have focused on cigarette smoking and excluded cigarillos, which are very similar to cigarettes in addictiveness and harmfulness [12]. This confusion has been exacerbated by conflation of ever‐use, past 30‐day use and ‘current’ use [11, 13].

Another example of confusing use of terminology is the term ‘e‐cigarette or vaping product use‐associated lung injury (EVALI)’ [14]. The term was adopted by the US Centers for Disease Control and others to describe a clinical condition involving acute lung damage from using certain types of vaping device [15]. It is built into the label that a cause of the condition is use of e‐cigarettes. However, the condition has been found not to be caused by use of e‐cigarettes *per se*, but almost certainly by vaping tetrahydrocannabinol (THC)‐containing e‐liquids adulterated with vitamin E acetate [14]. The widespread conflation of vaping THC with e‐cigarette use in this case has led to further inaccurate perceptions of the harmfulness of e‐cigarettes among members of the public [16]. Although an ontology may not immediately help with such issues, if scientists can be clear on the classification of products, so too may the writing of press releases, and other types of scientific communication to the press and the public. In particular, the ontology may help to highlight the distinction between harmfulness that arises from products themselves and that with their replaceable constituents such as different forms of e‐liquid.

Another area of confusion and lack of clarity are the terms used for inclusion criteria and outcomes in the research studied. For example, it is common for studies to conflate tobacco use, tobacco smoking and cigarette smoking. Therefore, randomised trials often refer to ‘smoking cessation’ as an outcome measure when the presumed meaning is cigarette smoking, tobacco smoking or even tobacco use. This may not appear to matter in cultures where cigarette smoking is the only form of smoking, but presents difficulties when attempting to integrate evidence internationally as in other locations or cultures cigarillos or other tobacco products may be more dominant.

Ontologies can go a long way to addressing such confusions. They are formal ways of representing the world using clearly delineated classes and their inter‐relationships, taking care to ensure that each entity is unique and unambiguously defined [17, 18]. Ontologies are being used in other areas of study such as biomedicine and social science to address problems of inconsistent and confusing terminology, for example, the Gene Ontology in molecular biology [19] and the Behaviour Change Intervention Ontology in behavioural science [20]. They provide a more secure foundation for advancing the science in a given domain, linking it with other domains, and making the output more usable by policy makers and practitioners.

The definitions of classes in ontologies differ from typical dictionary definitions. In ontologies, the definition is primary and the label is a way of referring to that defined entity, whereas in dictionary definitions the label is primary and the definition makes a claim about the correct usage of the label [17]. For example, in a dictionary definition one can claim that the correct meaning of the word ‘elephant’ is a large pachyderm with an elongated proboscis and large ears. In fields such as addiction, there is no unique ‘true’ meaning for a given term, because different people and organisations often use the same term to represent different things and use different terms to represent the same thing.

Use of ontological definitions is important because it avoids fruitless discussions about the ‘true’ meaning of labels and focuses instead on identifying classes of entities that can be unambiguously defined, whatever one chooses to call them. The label that is given to an entity is important, but only insofar as it allows users to make reference to entities without having to keep looking up the definition. The primacy of the definition allows users from different epistemological positions to take different views about what they mean by a label as long as they make it clear, which definition their preferred label relates to. This in turn means that a field can achieve wider convergence around a set of definitions even in areas where the use of specific labels is disputed. In ontologies, the concept of ‘namespace’ is used to uniquely specify the ontology to which a given label belongs, and within each ontology unique unambiguous identifiers specify entities. Therefore, if people want to use a term such as ‘dependence’ in different ways, they just need to specify the namespace and unique identifier for the term they are using to make clear what it is referring to. Each entity can then have different and potentially overlapping labels and synonyms to reflect different communities of usage.

The Society for the Study of Addiction has sponsored the initial development of an Addiction Ontology (namespace: AddictO) that can be applied across the whole addiction domain [21]. As part of this, and with additional funding from Cancer Research United Kingdom (UK) we are developing an E‐Cigarette Ontology (E‐CigO) to include all entities relating to e‐cigarettes and their study, which includes tobacco, nicotine and vaping products [4]. This paper reports the development of a part of E‐CigO concerning tobacco and nicotine products and vaping devices. The aim was to arrive at a set of definitions and labels that could be used to unambiguously characterise these products and show how they relate to each other. If this ontology becomes widely used it should improve research and practice in this area.

## METHODS

### Data sources for ontology development

The E‐CigO project was initiated with a webinar [22] hosted by Cancer Research UK (CRUK) in March 2019 (live event attended by ~60 individuals including academics, policy makers and stop smoking advisors/trainers). A subsequent in‐person meeting was held at University College London in May 2019 hosted by S.C., R.W., C.N. and J.H., attended by ~50 individuals with an academic and/or policy interest in nicotine and tobacco research, and 10 domain experts (representation from United Kingdom, United States and Europe, covering areas: policy, practice, research methods, public health, epidemiology, basic science and toxicology and special populations) who presented key areas of divergence within their specialist field. During these presentations the initial scope and range of terminology relevant for the e‐cigarette research domain was collected (by R.W.) and further developed through a snowball technique by group participation with those in attendance on the day. This initial workshop‐based scoping exercise led to ~700 terms being collated across the different aspects of e‐cigarette research and informed the main structure of the categorisation of the domain into modules, of which ‘products’, the subject of the present article, was one.

This scoping phase was followed by both top‐down and bottom‐up steps to progress the ontology development. In the bottom‐up step, a sample of the first 100 abstracts that were published in the *Addiction* journal within the previous year (2019) were reviewed, and all substantive terms were extracted and entered into a spreadsheet. These terms were separated into those that were generic (of relevance to the broader scientific domain) and those that were specific to the addiction domain. The resulting list of terms was merged with that arising from the workshop to create a master list of entities of relevance for the domain, with a flag to indicate those that were specific to the e‐cigarette domain in particular as opposed to those that were relevant for addiction in general, but not directly for the e‐cigarette domain (e.g. measures of alcohol dependence).

The top‐down step, which proceeded concurrently, involved an iterative process of attempting to identify sub‐domains of entities referred to in addiction papers. The results of this process will be reported elsewhere; for the current paper we note that one of the sub‐domains was labelled ‘product’ and this included all entities relating to products referred to in the addiction literature, including addictive products such as tobacco‐related products and alcoholic beverages, chemicals such as nicotine and alcohol and devices constructed to ingest chemicals.

**FIGURE 1:**
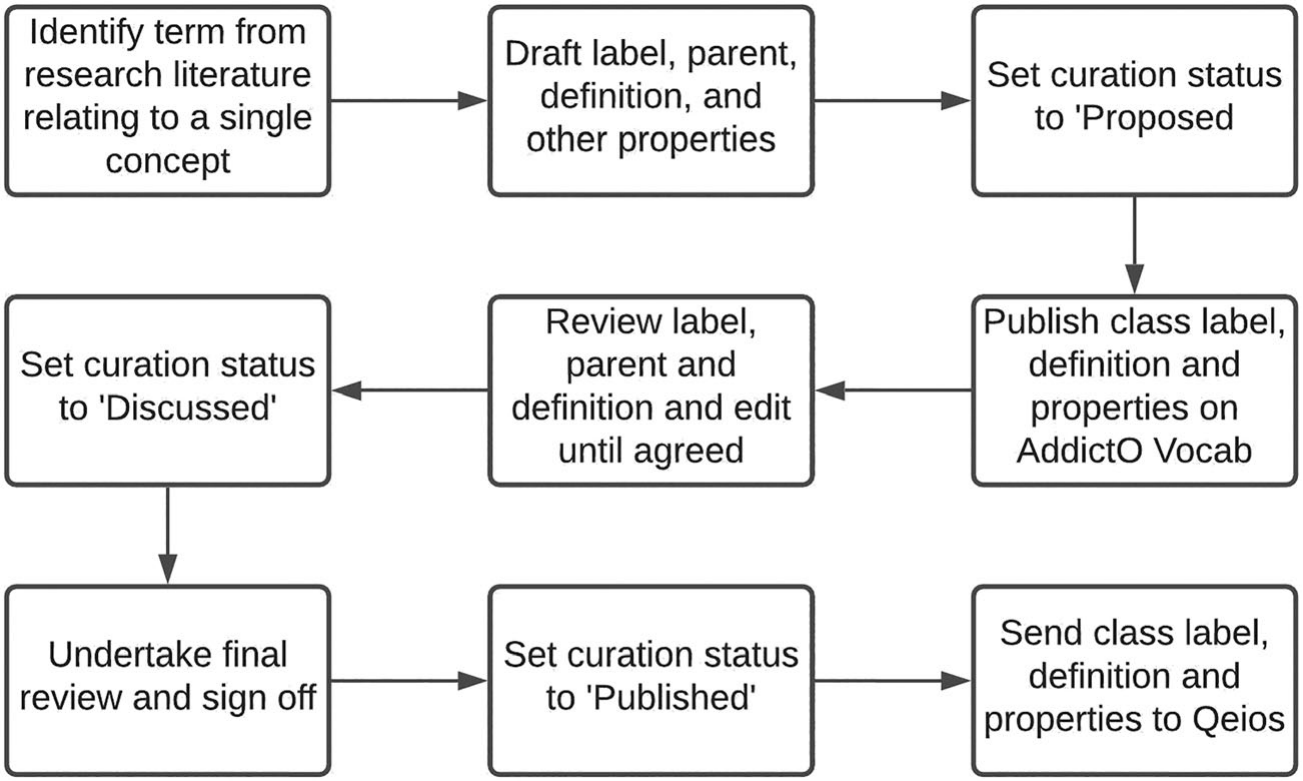
Workflow for including classes in the Addiction Ontology

The authors of this paper represent the core team who were responsible for curating the entities collected through the above processes and seeking agreement on what should be included or edited ready for inclusion. Each team member focused on a specific area to develop and sought feedback from the team at weekly meetings (held online) over a 2‐year period. This process allowed the team to systematically work through the entities by domain and agree on (i) whether the entity should be included, (ii) its position with the ontology, (iii) label and definition. We sought a majority consensus before including entities, if we could not seek agreement or the scope was beyond our group's expertise, we would seek input from an expert in that area or refer to other ontologies—although this only happened on a few occasions. J.H. was responsible for ontology expertise, consistency checks and assigning identifiers through automated processes. Figure 1 presents the workflow of the ontology from the point of agreed entity inclusion onward.

### Use of Basic Formal Ontology as the upper level ontology

A hierarchy of classes is a key feature of ontologies. As one goes up the hierarchy to more and more general classes, it is important for interoperability to connect up as far as possible with a single coherent set of upper‐most classes shared across domains. Most ontologies in biological sciences link to a single upper level ontology called Basic Formal Ontology (BFO) [23]. This was the upper‐level ontology chosen for AddictO and E‐CigO. An explanation of this and information about BFO can be found in our recently published paper [24].

In brief, BFO distinguishes two broad classes of entity: continuants (things that continue to exist as individuals over a period of time, such as objects and their attributes) and occurrents (things that take place or happen in time, such as processes). Within continuants, BFO distinguishes between ‘independent continuants’ such as objects and regions that have a physical presence and ‘dependent continuants’ such as qualities and dispositions that are attributes that have no separate existence beyond the things they are attributes of. ‘Qualities’ are attributes such as age and mass that are fully manifested in the things they are attributes of. Dispositions are attributes such as addictiveness and harmfulness that are more complex in that they are only conditionally manifested in certain contexts; they involve the potential for something to occur under certain conditions. A third important type of dependent continuant, that is not in BFO but is closely linked to it, is the ‘information content entity’. These are entities that provide information about things (e.g. ‘grams of tobacco’ provides information, using a unit of measurement, about an amount of tobacco).

Tracing all the classes in the ontology being developed to classes in BFO provides a basis for linking them to other ontologies that use BFO. In addition, AddictO and E‐CigO are developed using the technical standards advocated by the Open Biological and Biomedical Ontology (OBO) Foundry [25], which enable direct re‐use of ontology content across ontologies, avoiding duplication of effort. For example, classes representing chemicals such as nicotine do not have to be defined anew in AddictO, but can be imported from the Chemical Entities of Biological Interest (ChEBI) ontology, which adopts the same technical standards.

### Process of ontology development

A spreadsheet template was created with fields for all the properties of the entities and metadata about them. These included: ID (a unique identifier for the entity), label (a word or phrase representing the entity that is unique within the ontology), definition (an ontological definition of the entity specifying the parent class that it belongs to and features that distinguish this class from others in the parent class ) [17], informal definition (a less technical definition if required), logical definition (a formal expression defining the class in terms of a combination of other classes), a URL for a link to a source of the definition (e.g. Wikipedia) if there was one, parent class (the broader class to which it belongs), synonyms (alternative words or phrases used for this class, which do not have to be unique), comment (an elaboration and explanation of the definition if needed), curator note (an explanation if needed of the choices made in arriving at the label or definition) and curation status (a label to indicate whether the entity was still being worked on or was agreed).

The terms identified for inclusion were entered as labels into spreadsheets for each of the subdomains and the project team discussed and revised the labels, definitions and other fields until agreement was reached within the team. If there was a suitable entity that could be used from an existing ontology, this was imported; otherwise an entity was created. This is an ongoing process. Ontologies grow organically as new entities and relationships are added. Computer programs were written using the ROBOT tool to convert the spreadsheet representation into the standard format for ontologies, Web Ontology Language (OWL) [26].

Figure 1 shows the workflow for each class in the ontology, starting with a label and a preliminary definitions and proposed parent class. At this stage the class would be given a unique ID and its curation status would be ‘Proposed’. Next, it would be discussed within the team (all authors named above) and other information added as required, such as an informal definition, a comment to help interpret the definition and a curator note if required to explain to users why a particular definition or label was used. These fields would be reviewed and revised until there was agreement within the team. The curation status would then be changed to ‘Discussed’. There would be one further chance to review and amend before the curation status would be changed to ‘Published’. At this point, an automated process would transfer all the information for the class in the spreadsheet to the Qeios publishing platform where it would be published as a definition and assigned a digital object identifier (doi). Once the class was published on Qeios, there was an opportunity for anyone to comment publicly. Revisions could then be made and documented in response to those comments and an updated definition published on Qeios.

The Addiction Ontology under development is available for download in the OWL language from http://addictovocab.org/addicto.owl and the spreadsheets can be downloaded from the open source project repository at http://github.com/addicto-org/addiction-ontology. Classes that are under consideration or have been discussed or published can be explored at https://addictovocab.org/. Classes that have been agreed within the team are published on the Qeios publishing platform (www.qeios.com/) with a doi, which makes it easy to refer to them in articles using the standard crossref bibliographic referencing system (www.crossref.org). This paper reports only the major classes and relationships.

## RESULTS

### The tobacco and related products and their properties

**FIGURE 2:**
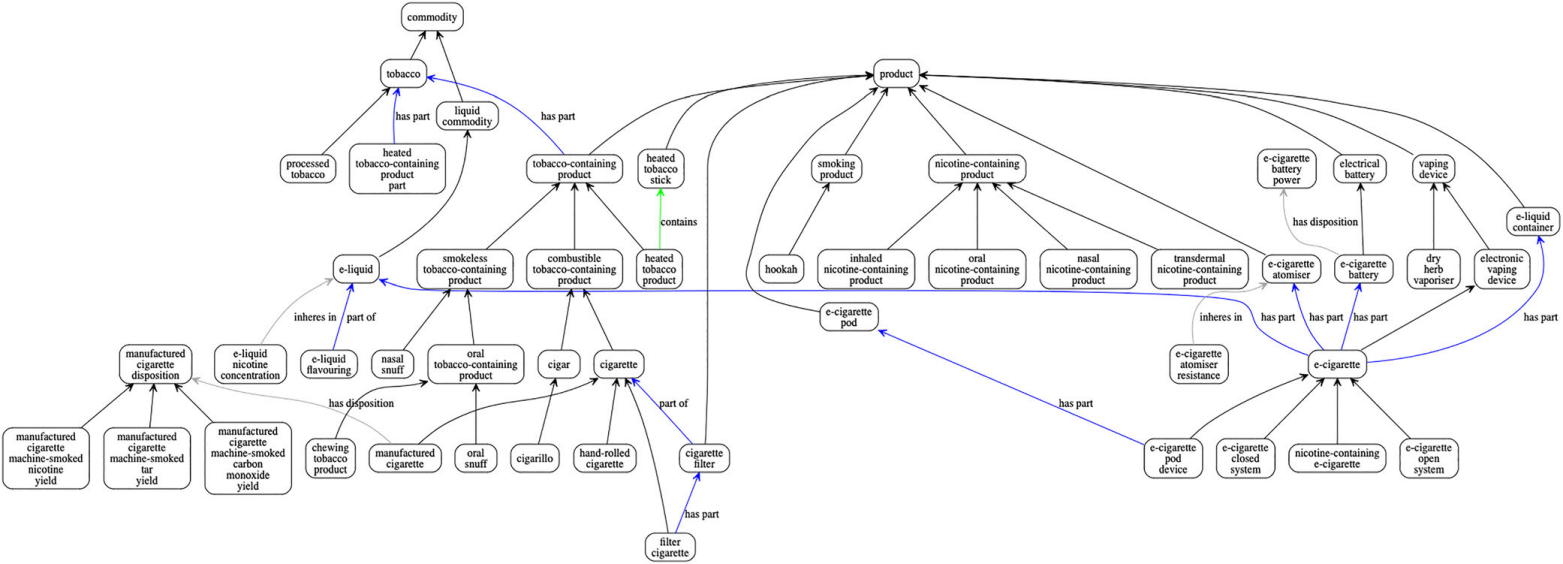
Major classes of tobacco and related products included in the Addiction Ontology. Each rounded box in the Figure represents a class in the ontology, whereas arrows represent relationships between classes. Black unlabelled arrows represent taxonomic (hierarchical) relationships; other relationships are labelled with the relationship type: ‘has part’ and its inverse ‘part of’ represent parthood relationships, ‘contains’ represents inclusion, ‘has disposition’ captures the relationship between a thing and the dispositional properties or attributes it has (properties that are only realized in certain contexts), and ‘inheres in’ captures the inverse relationship between a property or attribute and the thing it is a property or attribute of.

Figure 2 shows the major classes identified in the ontology and links these to classes in the BFO via another ontology called the Products Life Cycle Ontology (PLCO). The PLCO provides a way of linking tobacco products to other kinds of products [27].

Table 1 provides definitions of the entities and shows their parent classes. Full details of all the AddictO and E‐CigO classes can be found using the Qeios URL provided in the table.

**TABLE 1:** Major classes of tobacco and related products and their properties.

Label	Definition	Parent class	Link to details in Qeios
Upper‐level product entities			
Commodity	A material entity that bears a commodity role.	Material entity	www.qeios.com/read/Y31OI3
Commodity role	An economic good role that inheres in some material entity that is not an object and is realized in some act of appraisal	Economic good role	www.qeios.com/read/RGVIKK
Product	An artifact that has a product role.	Artifact	www.qeios.com/read/HC38WG.3
Product role	An economic good role that inheres in some artifact and is realized in some act of appraisal.	Economic good role	www.qeios.com/read/L0TR0T
Major classes of tobacco, nicotine and vaping products*			
Tobacco	A commodity that is formed from parts of the tobacco plant.	Commodity	www.qeios.com/read/R66G6A.2
Tobacco‐containing product	A product that is made of tobacco or has tobacco as a part and is used by people to ingest some tobacco constituents.	Product	www.qeios.com/read/X3XFMK.3
Nicotine‐containing product	A product that includes or contains nicotine for ingestion by the user.	Product	www.qeios.com/read/YH64Y3.2
Vaping device	A product that produces a vapour for inhalation by a person.	Product	www.qeios.com/read/VWA8JX
Electronic vaping device	A vaping device that produces vapour by electrically heating a substance that is an e‐liquid or a solid.	Vaping device	www.qeios.com/read/0NDEZK
Combustible tobacco‐containing product	A tobacco‐containing product in which the tobacco is processed to make it suitable for burning for the user to ingest the smoke.	Tobacco‐containing product	www.qeios.com/read/1CRCDI
Heated tobacco product	A tobacco‐containing product that contains a tobacco stick that it heats to cause minimal or no combustion to produce smoke or a vapour for inhalation by a person.	Tobacco‐containing product	www.qeios.com/read/WF7A8D.2
Smokeless tobacco‐containing product	A tobacco‐containing product in which the tobacco is processed for use in a manner that does not involve combustion.	Tobacco‐containing product	www.qeios.com/read/YN9XK1
Types of tobacco‐containing products			
Cigar	A combustible tobacco‐containing product that is a cylinder formed of chopped tobacco encased in tobacco leaf.	Combustible tobacco‐containing product	www.qeios.com/read/CKED6S.3
Cigarette	A combustible tobacco‐containing product that is a thin cylinder of finely cut tobacco encased in a thin paper.	Combustible tobacco‐containing product	www.qeios.com/read/CZN1E0.2
Bidi	A combustible tobacco‐containing product that is a thin small cylinder of dried leaf containing finely chopped tobacco.	Combustible tobacco‐containing product	www.qeios.com/read/342YIF
Oral tobacco‐containing product	A smokeless tobacco‐containing product that is manufactured to be held in the mouth.	Smokeless tobacco‐containing product	www.qeios.com/read/WCXQ3X
Chewing tobacco product	An oral tobacco‐containing product that is manufactured to be chewed.	Oral tobacco‐containing product	www.qeios.com/read/810RFA.3
Nasal snuff	A smokeless tobacco‐containing product that is formed of dry finely‐ground tobacco and is ingested by a person into the nasal cavity.	Smokeless nicotine‐containing product	www.qeios.com/read/T2DP55
Oral snuff	An oral tobacco‐containing product that is composed mainly or exclusively of moist, ground or powdered tobacco that is processed to make it suitable for use by a person placing it in the mouth between the gum and the cheek.	Oral tobacco‐containing product	www.qeios.com/read/6NCMYS.2
Cigarillo	A cigar that is short and thin with wrapping that is tobacco or tobacco‐based paper.	Cigar	www.qeios.com/read/SNP7WR
Manufactured cigarette	A cigarette that is manufactured in a factory.	Cigarette	www.qeios.com/read/XJ63ZR
Hand‐rolled cigarette	A cigarette whose parts have been purchased separately and assembled by hand or using a device for single assembly.	Cigarette	www.qeios.com/read/7UZ5V7
Types of nicotine‐containing products			
Oral nicotine‐containing product	A nicotine‐containing product that is manufactured to be held in the mouth or chewed so that nicotine is absorbed through the buccal mucosa.	Nicotine‐containing product	www.qeios.com/read/6IVS58
Nasal nicotine‐containing product	A nicotine‐containing product that is manufactured to enable users to ingest nicotine through the nasal mucosa.	Nicotine‐containing product	www.qeios.com/read/GSE0NT
Iinhaled nicotine‐containing product	A nicotine‐containing product that is manufactured to enable users to draw a nicotine‐containing vapour into the respiratory tract so that nicotine can be absorbed through the lining of this tract.	Nicotine‐containing product	www.qeios.com/read/XE3EUB
Transdermal nicotine‐containing product	A nicotine‐containing product that is manufactured to be attached to the skin so that nicotine can be absorbed through the skin.	Nicotine‐containing product	www.qeios.com/read/J10IA5
Types of e‐cigarette			
E‐cigarette	An electronic vaping device that is hand held and produces for inhalation an aerosol formed by heating an e‐liquid using a battery‐powered heating coil.	Electronic vaping device	www.qeios.com/read/0WX80U
Nicotine‐containing e‐cigarette	An e‐cigarette whose e‐liquid contains nicotine.	E‐cigarette	www.qeios.com/read/H51NZN
E‐cigarette open system	An e‐cigarette in which all of the main components are interchangeable.	E‐cigarette	www.qeios.com/read/ROMCLB.2
E‐cigarette pod device	An e‐cigarette that has as a part a replaceable e‐cigarette pod.	E‐cigarette	www.qeios.com/read/G3SA5I.2
E‐cigarette closed system	An e‐cigarette in which none of the main components is interchangeable.	E‐cigarette	www.qeios.com/read/6CURTR.2
Manufactured cigarette attributes			
Manufactured cigarette machine‐smoked nicotine yield	A disposition of a manufactured cigarette to produce a quantity of nicotine in the smoke when tested in a smoking machine that is programmed to take puffs in a standard way.	Manufactured cigarette disposition	www.qeios.com/read/H66X12
Manufactured cigarette machine‐smoked tar yield	A disposition of a manufactured cigarette to produce a quantity of tobacco particles in the smoke produced when tested in a smoking machine that is programmed to take puffs in a standard way.	Manufactured cigarette disposition	www.qeios.com/read/Z8I4KV
Manufactured cigarette machine‐smoked carbon monoxide yield	A disposition of a manufactured cigarette to produce a quantity of carbon monoxide in the smoke produced when tested in a smoking machine that is programmed to take puffs in a standard way.	Manufactured cigarette disposition	www.qeios.com/read/HLY8QY
Tobacco product parts			
Cigarette filter	A product that is manufactured to be part of a cigarette, located at the end that the user puts to the lips, which blocks some smoke particles from entering the mouth during use.	Product	www.qeios.com/read/KHDM2E.2
Heated tobacco stick	A product that is formed of tobacco processed in such a way as to make it suitable to be used in a heated tobacco‐containing product.	Product	www.qeios.com/read/Y8N3SW.2
E‐cigarette product parts and characterisation			
E‐cigarette atomiser	A product that is manufactured to be part of an e‐cigarette and to heat e‐liquid during e‐cigarette use to produce a vapour.	Product	www.qeios.com/read/Z5UFSA.2
E‐liquid container	A product that is manufactured to be part of an e‐cigarette and contain e‐liquid.	Product	www.qeios.com/read/AE8840
E‐cigarette pod	A product that is manufactured to be part of an e‐cigarette as a combination of a container for e‐liquid, an atomiser and systems for controlling the atomiser connected together into a single component.	Product	www.qeios.com/read/PRF1L8.2
E‐cigarette battery	An electrical battery that is manufactured to be part of an e‐cigarette and provide electrical energy for an e‐cigarette atomiser.	Electrical battery	www.qeios.com/read/04EHV4
E‐liquid	A liquid commodity that is used in e‐cigarettes for the purpose of aerosolization and inhalation by a person.	Liquid commodity	www.qeios.com/read/K8QA62.2
E‐liquid nicotine concentration	The concentration of nicotine in a portion of e‐liquid.	Concentration	www.qeios.com/read/Y3OE0H
E‐liquid flavouring	A chemical substance that is designed to be part of an e‐liquid to impart a flavour to the vapour produced when the e‐liquid is used in an e‐cigarette.	Chemical substance	www.qeios.com/read/GRDC8W.2
E‐cigarette battery power	A e‐cigarette battery disposition to transfer electrical energy in a circuit containing an atomiser.	E‐cigarette battery disposition	www.qeios.com/read/24QQDS
E‐cigarette atomiser resistance	An e‐cigarette atomiser quality that is the opposition of its circuit to the flow of current in that circuit.	E‐cigarette atomiser quality	www.qeios.com/read/ZIM0VV

*Note*: Ontological definitions can seem technical and are sometimes not easy to read. Informal definitions are being added to AddictO Vocab to address this; however, informal definitions should always be read only alongside the formal definition and not in isolation to avoid the risks associated with ambiguity. Some internet browsers direct users to the Qeios landing page and not directly to the definition.

* Major classes are not always informative because they cover a variety of products.

AddictO distinguishes tobacco‐containing products as products that consist of or include tobacco that are distinct from nicotine‐containing products that in turn are distinguished from vaping devices. This upper‐level distinction is essential to ensure consistency and clarity in the use of terms. Therefore, although nicotine transdermal patches may contain nicotine, they do not contain tobacco, although they are used to ingest a chemical, nicotine, which is usually (but not always) found in tobacco. Similarly vaping devices cannot be classified as either nicotine‐ or tobacco‐containing products because in many cases they contain neither nicotine or tobacco, or indeed a psychoactive substance of any kind.

Entities that the US FDA calls ‘tobacco products’ had to be considered as a separate class because of the inconsistencies in the definition. This class is not included in Table 1 or Fig. 2, but can be found on Qeios (www.qeios.com/read/VMLSA4.4). Although this definition has been developed for purposes of regulation in the United States, its potential for causing confusion means that it is better avoided in scientific discourse unless the purpose is to refer specifically to the regulatory status of a product in the United States.

To define tobacco‐containing products, we have to define tobacco and assign it a parent class. An obvious parent class would be ‘psychoactive substance’, but biomedical ontologies typically do not use psychological or biological effects of chemicals as their primary basis for classification of substances because these are complex and context‐specific attributes. Therefore, ‘nicotine’ in the CHEBI ontology is classed according to its chemical structure. Another reason for not using psychological or biological effects in the primary classification is that any chemical or substance may have many such actions. Nicotine is not only psychoactive, it has effects on the cardiovascular and many other body systems. Therefore, we used commodity as the parent for tobacco and product for the parent for tobacco product.

The ontology subdivides tobacco‐containing product classes into combustible tobacco‐containing products, heated tobacco products and smokeless tobacco‐containing products. This is to reflect the most common upper‐level division of these classes of product in common usage. It also captures some empirical distinctions. Combustion of tobacco creates additional toxins that increase their harmfulness to health. Combustible products are also designed for users to inhale the smoke, which offers both the potential for increased harm because of toxic chemicals coming into contact with the large and delicate tissues of the lungs and upper airways, and more rapid ingestion of nicotine, which is the primary psychoactive compound in tobacco.

Combustible tobacco‐containing products come in many forms. The ontology, based on common usage, distinguishes between cigars (including cigarillos) whose outer covering is made from tobacco, cigarettes (whose outer covering is made from a thin paper), and bidi (which are like small cigarettes except that their outer covering could be made from paper or a processed plant leaf). It is important to note that beyond this distinction in terms of components, the classes of combustible tobacco‐containing products provide little information on attributes of interest, particularly harmfulness and addictiveness.

This severely restricts the value of relying on these classes per se as a basis for clinical or policy decisions. For example, cigarillos of the type widely used in the United States are classified as cigars, but in terms of harmfulness and addictiveness have much more in common with cigarettes than with large panatella cigars because of key attributes that influence the way they are used [28]. In particular, the smoke is readily inhaled into the lungs and they are cheap and convenient to use.

An important distinction between types of cigarettes is between manufactured cigarettes and hand‐rolled cigarettes. This distinction is important because the former can be characterised in terms of machine‐smoked nicotine yield, machine‐smoked tar yield, and machine smoked carbon monoxide yield, whereas the latter cannot. The latter are typically much less expensive than the former. Note that machine‐smoked yields of cigarettes bear very little relation to the amount of nicotine, tar and carbon monoxide that users typically obtain from them because of compensatory puffing and inhalation patterns [29]. Nevertheless, they need to be represented in the ontology because they are often referred to in research and policy.

A separate major class had to be created for heated tobacco products such as iQos. This is because, although it has been claimed that there is no combustion, they produce carbon monoxide, which indicates that the temperature attained does lead to some combustion. Moreover, they cannot be considered combustible tobacco‐containing products because the tobacco stick that is heated is not consumed by the heat. They could potentially be considered as a vaping device, but they do contain tobacco and so it was judged that it would be best to classify them as tobacco‐containing products.

Smokeless tobacco‐containing products come in many forms that are distinguished in common usage by their intended route of administration: primarily oral versus nasal. They can also be distinguished by the way that the tobacco used in them is processed because this has implications for their harmfulness. Snus is processed in a way that results in very low concentrations of tobacco‐specific nitrosamines and this is reflected in their low carcinogenicity relative to, for example, chewing tobacco of the kind used in the United States. However, as with combustible tobacco, this broad classification is of limited use for policy and clinical decision‐making and needs to be supplemented by information about specific attributes of individual products.

There are a very large number of smokeless tobacco‐containing products in use around the world and we are working with experts to include as many of these as possible in the ontology. This is work in progress and will be reported separately.

Nicotine‐containing products vary by route of nicotine absorption, intended method of administration, rate of nicotine absorption, and nicotine dose. In common usage products are classified by their intended route of administration: oral‐buccal (held in the mouth or chewed), oral‐alimentary (swallowed), pharyngeal‐pulmonary (inhaled), nasal (sniffed or placed in the nasal cavity) and transdermal (attached to the skin).

Tobacco‐containing products are typically, although not necessarily, nicotine‐containing products, but many nicotine‐containing products are not tobacco‐containing products. The route of administration provides some information about the rate of nicotine absorption that can be expected with transdermal being the slowest and pharyngeal‐pulmonary being the fastest. This in turn may provide information on their addictiveness [30]. However, individual products may differ considerably in these attributes so class membership cannot be used definitely for this inference. It should also be noted that the route of administration can allow inferences to be drawn about the rate of nicotine absorption. For example, the transdermal route produces slower absorption than the buccal route, which provides slower absorption than the nasal route, which provides slower absorption than the pulmonary route [30].

Vaping devices can be further classified according to whether the heat needed to vaporise the material is provided electronically or through combustion. Therefore, we can identify electronic vaping device and a subclass of this, the e‐cigarette. Not all vaping devices are electronic and not all electronic vaping devices are e‐cigarettes. Moreover, not all e‐liquids used in e‐cigarettes contain nicotine. For the reasons above, when describing use of these devices it is essential to make distinctions between the different products.

### E‐cigarettes and their properties

E‐cigarettes have evolved rapidly and the terminology used to label them is variable and often imprecise. The major distinctions appear to be between devices that have no replaceable components (e‐cigarette closed systems) and those that have replaceable components: the battery, atomiser (that heats the e‐liquid), the tank (liquid container) or the e‐liquid. There are also e‐cigarette pod devices in which the atomiser and e‐liquid container form a single part (the pod), which can be replaced by one with a similar design.

Another important distinguishing feature is the use of flavourings in the e‐liquid. The large number of combinations of features that may be important when characterising the effects of e‐cigarette use means that it is better to characterise e‐cigarettes by specific features than by broad classes.

The resistance of the atomiser, power rating of the battery, nicotine concentration of the e‐liquid and choice of flavourings appear to be important features that should be represented. Figure 2 and Table 1 show the proposed classes for use with e‐cigarettes. The Addiction Ontology specifies that e‐cigarettes have as parts batteries, atomisers and e‐liquid containers, which contain e‐liquid, and then specifies the properties of these. This provides a systematic way of capturing the complexity of the differing properties of e‐cigarettes, which in turn can provide a basis for gathering data on these properties. More classes that capture different variants of e‐cigarettes can be found on AddictO Vocab (addictovocab.org).

Given the varied ways in which tobacco products are labelled in research reports and policy documents, the proposed ontology includes synonyms for some of the classes. Synonyms do not have to be unique, but provide a way of searching the ontology for classes based on colloquial terms. These synonyms can be found in AddictO Vocab.

## DISCUSSION

Creating an ontology of tobacco and related products involves making important distinctions between classes that, although they may be related in practice, have to be treated as separate. Tobacco‐containing products cannot logically include nicotine‐containing products, and vaping devices are a separate class. Each of these classes has subclasses that need to be distinguished from each other in research reports and policy documents. The ontology proposed in this paper makes in‐roads to achieving this. The definitions are published on Qeios and readers are invited to comment.

Adopting the terms and definitions in the ontology should help to avoid researchers and policymakers confusing vaping with e‐cigarette use, cigarette smoking with tobacco smoking and nicotine use with tobacco use. However, this will not happen overnight and there is a role for journals and research funders to foster good practice.

Researchers can immediately begin to use the ontology by using the labels in their research reports and referring to the definitions in Qeios. Journals and research funders can begin to set standards when it comes to use of terminology to help ensure clarity. Researchers and practitioners can also join the ongoing discussion about the terms and definitions and develop expertise in ontologies to improve and extend the ontology.

Work is currently underway to extend the ontology to the wide range of smokeless tobacco‐containing products in use around the world. There is also ongoing work to define and label e‐cigarette components, and to use the ontology of products to define tobacco and nicotine use behaviours [4].

It is important to note that the ontology definitions presented here are based on the product characteristics and what they are designed to do by the manufacturer. That is, although an e‐cigarette may be used by a sub‐community for purposes other than vaping nicotine (e.g vaping illicit substances [30]) the products were not designed for this purpose. Indeed, in this case, it is actually clearer for the reader if they understand that another substance has been used within an e‐cigarette for purposes that the product was not designed for, rather than trying to shape the definition of a device to accommodate all the possibly diverging ways it is being used in practice. Problems arise when researchers create their own labels for products (e.g. alternative nicotine delivery systems [ANDS]) or are vague as to the product characteristics.

However, for completeness and to make the resource useful and searchable we include product synonyms. Synonyms provide a useful searchable point of reference, but the primary product label should be used wherever possible. This is because synonyms (e.g. vape as a synonym for e‐cigarette), although familiar to some researchers, are often based on colloquialisms and do not reflect the object as either it truly is or how the manufacturer designed the product to be used. As mentioned before, within this ontology synonyms are not always exact equivalences, because they may also offer the ontology user the opportunity to see how the product is being broadly referred to even if those terms are ambiguous. An example of a synonym that is not an exact equivalence is e‐cigarette and electronic nicotine delivery device (END). A further example of a synonym that is a colloquial term is when referring to e‐cigarette use as ‘non‐cigarette tobacco use’ [31]. This expression is factually incorrect as e‐cigarette use is not tobacco use, but could nevertheless be included within the ontology as a synonym connected to the class ‘e‐cigarette use’.

Retroactively, the ontology can be used to synthesise previously published data, to understand how findings in relation to different products align, depending on how the products are described (if at all), fit within these categories and where errors with the published data may exist. For example, in reviews of the literature on product use and health impacts it is important to separate the different types of e‐cigarette and tobacco product, before it is possible to draw integrated conclusions about their respective health impacts. The ontology, therefore, provides a vital resource for systematic reviews, living reviews, meta‐synthesis and literature scoping reviews.

Limitations of the organisation of definitions in an ontological structure need to be acknowledged. Tobacco, nicotine and vaping research is a broad field encompassing many products that are continuously evolving. The taxonomy presented here is not complete and it is possible that because of the limitations of our scoping exercises some domains and entities have been missed. Ontologies are themselves living entities, continuous works in progress, and we refer readers to http://addictovocab.org/addicto.owl for the most up‐to‐date version. New products and more detail will continually be added to the ontology; therefore, lower level sibling relationships and the level of detail may change as new information becomes available, although the main hierarchy and distinctions between these products will most likely not change dramatically. The ontology and its components are subject to open peer review and public involvement that we expect to add greater depth, clarity, granularity and contextualisation to our representation of products.

## CONCLUSIONS

An ontology is proposed that makes important distinctions between tobacco‐containing products, nicotine‐containing products and vaping devices. It identifies subclasses of these and properties of these subclasses. We have reported an initial version of the ontology. The aim is that the research and practitioner community can make use of the opportunities provided to comment on and help to extend the ontology and that journals and funders will encourage and support researchers to use the ontology to improve clarity in their writing.

## DECLARATION OF INTERESTS

S.C. is a Senior Editor at Addiction. R.W. is an unpaid director of the Unlocking Behaviour Change Community Interest Company. He has undertaken research and consultancy for companies that manufacture licensed smoking cessation medicines (Pfizer and GSK). C.N. is an Associate Editor for Addiction. K.S. and J.H. have no competing interests.

## AUTHOR CONTRIBUTIONS

**Sharon Cox:** Conceptualization; data curation; formal analysis; funding acquisition; investigation; project administration. **Robert West:** Conceptualization; data curation; formal analysis; funding acquisition; investigation; methodology; project administration. **Caitlin Notley:** Conceptualization; formal analysis; funding acquisition; investigation; project administration. **Kirstie Soar:** Data curation; formal analysis; investigation; project administration. **Janna Hastings:** Conceptualization; data curation; formal analysis; funding acquisition; investigation; methodology; project administration; resources; software; visualization.
